# Human resources for health at the district level in Indonesia: the smoke and mirrors of decentralization

**DOI:** 10.1186/1478-4491-7-6

**Published:** 2009-02-03

**Authors:** Peter F Heywood, Nida P Harahap

**Affiliations:** 1 Australian Health Policy Institute, University of Sydney, Sydney, NSW, Australia; 2 Jalan Bukit Dago Selatan, Bandung, West Java Province, Indonesia

## Abstract

**Background:**

In 2001 Indonesia embarked on a rapid decentralization of government finances and functions to district governments. One of the results is that government has less information about its most valuable resource, the people who provide the services. The objective of the work reported here is to determine the stock of human resources for health in 15 districts, their service status and primary place of work. It also assesses the effect of decentralization on management of human resources and the implications for the future.

**Methods:**

We enumerated all health care providers (doctors, nurses and midwives), including information on their employment status and primary place of work, in each of 15 districts in Java. Data were collected by three teams, one for each province.

**Results:**

Provider density (number of doctors, nurses and midwives/1000 population) was low by international standards – 11 out of 15 districts had provider densities less than 1.0. Approximately half of all three professional groups were permanent public servants. Contractual employment was also important for both nurses and midwives. The private sector as the primary source of employment is most important for doctors (37% overall) and increasingly so for midwives (10%). For those employed in the public sector, two-thirds of doctors and nurses work in health centres, while most midwives are located at village-level health facilities.

**Conclusion:**

In the health system established after Independence, the facilities established were staffed through a period of obligatory service for all new graduates in medicine, nursing and midwifery. The last elements of that staffing system ended in 2007 and the government has not been able to replace it. The private sector is expanding and, despite the fact that it will be of increasing importance in the coming decades, government information about providers in private practice is decreasing. Despite the promise of decentralization to increase sectoral "decision space" at the district level, the central government now has control over essentially all public sector health staff at the district level, marking a return to the situation of 20 years ago. At the same time, Indonesia has changed dramatically. The challenge now is to envision a new health system that takes account of these changes. Envisioning the new system is a crucial first step for development of a human resources policy which, in turn, will require more information about health care providers, public and private, and increased capacity for human resource planning.

## Background

In 2001 Indonesia embarked on a rapid decentralization of government finances and functions [1]. Within a year, much of the responsibility for public services had been assigned to the districts: more than 70% of central civil servants, as well as most service facilities, were transferred to the local governments. In parallel, Indonesia also commenced implementation of a new intergovernmental fiscal framework; the apparent district share in government spending almost doubled; and the balance between general grants and grants earmarked by the centre for specific sectors and functions seemed to change markedly in favor of general grants, the sectoral allocation of which was to be decided by local government. However, because it happened so quickly, there was still much that remained to be done. In some cases implementing regulations have still not been completed; in others there is conflict, ambiguity and confusion between the various laws and regulations. As a result, more than eight years later, uncertainty still affects the efficiency of service delivery.

As outlined by Bossert [2], the underlying notion of decentralization "...implies the expansion of choice at the local level." Using a principal/agent approach, Bossert describes this expansion as "decision space", "the range of effective choice that is allowed by the central authorities (the principal) to be utilized by local authorities (the agents)." The notion of decision space can then be used to assess the situation for the various functions and activities of local authorities. Viewed in this way, decentralization is a process, the outcome of which may vary across functions and over time.

Consistent with this approach, the radical and rapid change in intergovernmental relations in Indonesia was expected to lead to many changes at the district level, especially to improved public sector performance. These expectations were based on the view that although districts would remain heavily dependent on transfers of funds from central government for their revenue, the tight specification of the way in which funds would be used, which characterized the highly centralized government of the Suharto era, would be greatly relaxed and the districts would now decide how funds would be spent – this increased autonomy at the local level was then expected to result in decisions more suited to the local setting and improved outcomes.

Like other government services, the health sector has also been affected by these changes. One of the areas in the health sector most affected is human resources. Prior to decentralization, the central Ministry of Health had complete responsibility for the health sector, including human resources, and decided how resources were to be allocated in the districts. Although in principle the districts now have control of their public sector health workforce (Hence the statement in one important analysis of decentralization in Indonesia [1] that 'Over 2 million civil servants, or almost two thirds of the central government workforce, were transferred to the regions.'), the central government still controls all permanent civil servants (*Pegawai negeri sibil *– PNS, see Additional file. 1) working at the district level; these staff are paid directly from the centre and the centre effectively controls hiring, firing and the conditions of employment of this category of staff. The centre also controls hiring, firing and the conditions of employment of a category of contract staff known as PTT (*Pegawai Tidak Tetap *– see Additional file. 2).

However, there are, in addition, many public sector staff members contracted at the district level who are neither PNS or PTT. These locally contracted staff have been crucial to allowing districts to develop flexibility in total numbers and skills mix in their staffing plans. The central government has little, if any, information about this category of staff – their qualifications, how many there are, where they work or the conditions of their employment.

Before decentralization, districts were obliged to respond to demands from the central government for information about use of resources, health status, the delivery of services and human resources for health. Although there were inaccuracies in the data and delays in receipt at the center, it was possible for the central government, through their representatives in the provinces and districts, to build a picture of the situation at the district, provincial and national levels. With decentralization the districts no longer feel as obliged to maintain these records or to respond to requests for information from the center. In addition, there is an increasing number of private sector health care providers who do not work for the government at all, and the central government has little information about them as well. Consequently, one of the effects of decentralization is that the centre now has less information for the sector as a whole about its most critical asset, human resources, than it did before. And this is occurring at a time when the there is great concern about the lack of attention to human resources in the health sector globally, especially that many governments do not have even basic information about their most important resource: how many health professionals, their age and sex, or how they are distributed [3]. At the same time, there are clear indications that the health system and the health needs of the population are changing and that government must modify policies in response to these changes and shape a health system that can cope with the future. Reliable information about human resources for health is vital to envisioning a health system that can respond to the health challenges facing Indonesia.

The work reported here is part of an attempt to understand what is happening at the district level in the health sector, starting with a basic enumeration of the human resources and the health facilities in which they work and deliver services. Our aim, in a sample of 15 districts in Java, is to: (1) enumerate the stock of health facilities (public and private) in the health sector in 2006; (2) enumerate the stock of human resources (public and private) in the health sector in 2006 trained to provide care and treatment for illness – in Indonesia this means doctors, nurses and midwives; and (3) estimate the funds (public and private) spent on health care in the course of 2006. The results will be reported in separate papers. This paper reports on human resources for health and aims to address the following questions:

• What is the stock of human resources for health trained to provide care and treatment for illness (doctors, nurses and midwives) at the district level, by professional group?

• What is the service status of these health care providers at the district level?

• What is the primary place of work of these health care providers at the district level?

• What was the effect of decentralization on human resources for health at the district level?

• What are the implications of the results for future development of the health sector?

## Methods

As much of the information we wished to obtain is not available at the central Ministry of Health, we collected it in the districts. This work concentrates on Java, where 60% of the Indonesian population lives. Resources were sufficient to allow data to be collected in 15 districts. To ensure representation of the range of situations in Java, five districts were chosen in each of three provinces: West Java, Central Java and East Java. Basic details of the 15 districts are shown in Table 1.

**Table 1:** Estimated 2006 population of 15 districts included in this study

**Province**	**District**	**Population**	**Number of subdistricts**
West Java	Ciamis	1458680	36

	Cirebon	2134656	37
	
	Garut	2274973	41
	
	Subang	1402134	22
	
	Sukabumi	2240901	45

Central Java	Brebes	1727708	17

	Cilacap	1717273	24
	
	Jepara	1078037	14
	
	Pemalang	1341422	14
	
	Rembang	591786	14

East Java	Jombang	1203716	21

	Ngawi	857449	19
	
	Pamekasan	782917	13
	
	Sampang	801541	14
	
	Trenggalek	682328	14

Data were collected by three teams, one for each province, in 2007. The provincial team leaders were from, and based in, the province, and had previous experience in collecting health data at the district level.

The goal was to enumerate all health care providers (doctors, nurses and midwives) in the district by professional qualification, service status and primary place of work. The primary source of data on district health personnel was the district health office and the district hospital. There are two basic documents usually available at each district health office and district hospital – a list of all government employees in the sector by rank and seniority (*Daftar Nominatif*), and the list of all permanent civil servants in the district by sector (*Daftar Urut Kepangkatan*, also known as the *DUK*). All health care providers who do not work for the government but have a private practice in which health care is provided should be licensed by the district government; our list was supplemented from those sources as well.

While these lists were kept more or less up to date in the past, since decentralization many districts put much less effort into these tasks. Consequently there is considerable variation between districts (and provinces) in the completeness of these lists today. In some districts where the government records were clearly incomplete, we also consulted the membership lists from the professional associations for doctors, nurses and midwives – these lists potentially include members in both the public (because public sector doctors, nurses and midwives are members of the associations) and private (because doctors and midwives have private practice rights) sectors and are also in varying states of completeness.

Regardless of the source of information, all names on the membership lists were checked against the public sector lists to minimize double counting. Thus, a consolidated list of doctors, nurses and midwives (see Table 2 for definitions) was produced for each district. For each provider we also recorded their employment status (civil servant, contract, volunteer, self employed – see Table 3 for a list of categories and definitions) and primary place of work (hospital, health centre, private practice, clinic – see Table 4 for a list of categories and definitions). In West Java this information is essentially complete. In the other two provinces, East Java and Central Java, there were districts in which the information on each provider did not include employment status and/or primary place of work. The aggregate information on employment status and primary place of work for the districts in these provinces is based on information available in the annual district health sector report and discussions with senior administrators in the district health office.

**Table 2:** Definitions of health service providers

**Provider**	**Description**
Doctor (Dokter)	Graduate of an Indonesian medical school licensed by the government.

Nurse (Perawat)	Graduate of:
	(1) a Sekolah Perawat Kesehatan (SPK): students enter at the end of junior high school and the SPK training is regarded as equivalent to senior high school; or
	(2): an Akademi Perawatan for which students enter at the end of senior high school; or
	(3): Fakultas Ilmu Keperawatan, a university-level course at the first degree level; there are a small number of second degree-level graduates as well. All these institutions must be licensed by the government.

Midwife (Bidan)	Graduate of:
	(1) Sekolah Bidan (SB): students enter at the end of junior high school and this training is regarded as equivalent to senior high school; or
	(2): Program Pendidikan Bidan (PPB) – entrants to this one-year programme have an SPK nursing qualification; or
	(3) Akademi Kebidanan (Akbid), which students enter at the end of senior high school.
	Originally midwives were trained as SB until this programme was closed in 1984. After a five-year period of no training of midwives, the government started training again in 1989 through the PPB as village midwives; the PPB was closed in 1998 and was replaced by the Akbid programme.

**Table 3:** Categories of employment status of health service providers (doctors, nurses, midwives)

Status	Category		Employer
Permanent civil servant	PNS	Central government	See Additional file. 1.

Central contract	PTT	Central government or, in the case of a small number of doctors, local government.	See Additional file. 2.

Local contract	*Kontrak/honorer*	Local government, health facility using funds from the local government.	Doctor, nurse or midwife who works for a health facility on a local government contract. The level of pay and terms are usually less favourable than those for a PTT. Paid, hired and fired by the district government from its own budget. Terms and conditions of their employment are not well documented, but there seems to be variation between facilities and districts.

Volunteer	*Sukwan*	Health facility using locally generated funds.	Doctor, nurse or midwife who works as a "volunteer" at the health facility under a short-term informal "contract". They receive some payment directly from the facility and usually hope that their work as a volunteer will eventually lead to a longer-term contract and/or PNS.

Monthly contract	*Bidan harian lepas*	Health facility using funds provided by the province.	Village midwife employed on a monthly basis. This category of provider is used only in West Java Province since 2005.

Private practice	*Praktek swasta*	Self	Doctors, nurses or midwives who work primarily on their own account as private practitioners and do not have a primary appointment with, or receive a salary from, the government. This category does not include doctors and midwives whose primary appointment is with the government but who also have a private practice after office hours.

**Table 4:** Definitions of health facilities*

**Health facility**	**Description**	**Public/Private**
Public hospital (*Rumah Sakit Umum Daerah *(RSUD))	Public hospital located at the district level.	Public

Private hospital (*Rumah Sakit Umum Swasta *(RSUS))	Private hospital located at the district level, national and provincial government enterprises, police, defense forces.	Private

Private hospital for women and children (*Rumah Sakit Ibu dan Anak *(RSIA))	Private hospital for women and children located in the district.	Private

*Rumah Sakit Bersalin *(RSB)	Private women's hospital located in the district.	Private

Private maternity clinic (*Rumah Bersalin *(RB))	Private maternity clinics with more than two beds.	Private

Health Centre (*Pusat Kesehatan Masyarakat*)	Public health centre. In general they are located at the subdistrict level.	Public

Auxiliary health centre (*Pustu*)	Public health subcentre – in general they are located at the subdistrict level, usually in a village.	Public

Village midwife (*Bidan di desa (BDD*)/*Pondok Bersalin Desa (Polindes*))	BDD is a village midwife who receives a government salary and also may charge for the services she provides and retain the feef. Although the village midwife theoretically lives in the village (*desa*), there are reports that in many villages she lives elsewhere, maybe in a nearby urban area. The services provided by the BDD may be offered in a room in her house or in a structure in that is the property of, and was built by, the village government (*polindes*). In the *polindes *the services are provided by the village midwife, who charges for the services and retains the fees.	Private

Treatment clinic (*Balai pengobatan *(BP))	Treatment clinic. Before the advent of the health centre, there were private and public treatment clinics. As the health centre was developed, the public treatment clinics were incorporated into the health centres, with the result that only the private *balai pengobatan *remained. Although they have been ignored by the government and donors, they remain a significant source of treatment, especially in urban areas. They are licensed by the local government and must have a doctor as the supervisor. In practice, most of the doctors named as the supervisor seldom visit and nurses, and some midwives, provide most of the health care unsupervised.	Private

Doctor, private practice (*Dokter praktek swasta (DPS) murni*)	Doctor whose primary professional activity is private practice and who does not receive a salary from the government.	Private

Nurse, private practice (*Perawat praktek swasta (PPS) murni*)	Nurse whose primary professional activity is private practice and who does not receive a salary from the government.	Private

Midwife, private practice (*Bidan praktek swasta (BPS) murni)*	Midwife whose primary professional activity is private practice and who does not receive a salary from the government.	Private

*A health facility is defined as a physical structure that varies from a large complex of buildings to a single room in a house from which health services are offered by a doctor, nurse or midwife.

## Results

The results provide a snapshot of the human resource situation in the health sector in 2006 for 15 districts across Java.

### Density of health care providers

The totals for doctors, nurses and midwives for each district are shown in Table 5. There is an almost fourfold range in the total number of health care providers across these 15 districts, from 515 in Sampang to 1818 in Cirebon. As would be expected, there is a high correlation (r = 0.85) between district population and total health care providers. On average the number of providers increases by 300 for every 500 000 increase in population. However, when the number of providers in a district is expressed in terms of population (provider density, total number of providers per 1000 population – see Table 6), the provider density shows a negative correlation with district population (r = -0.46): districts with larger populations tend to have lower provider density. While this may be a reflection of some economies of scale, there may be other issues here that our data cannot address (for example, the surface area and population density of the districts).

**Table 5:** Total doctors, nurses and midwives in 15 districts by province and district. 2006

**Province**	**District**	**Doctor**	**Nurse**	**Midwife**	**Total**
West Java	Ciamis	96	835	472	1403

	Cirebon	295	800	723	1818

	Garut	145	984	468	1597

	Subang	173	751	442	1366

	Sukabumi	206	588	406	1200

Central Java	Brebes	181	599	548	1328

	Cilacap	183	873	585	1641

	Jepara	130	552	383	1065

	Pemalang	130	519	313	962

	Rembang	92	329	425	846

East Java	Jombang	301	577	408	1286

	Ngawi	132	446	203	781

	Pamekasan	87	299	253	639

	Sampang	53	291	171	515

	Trenggalek	73	358	216	647

**Table 6:** Provider density (per 1000 population) for doctors, nurses and midwives in 15 districts by province and district, 2006

**Province**	**District**	**Doctor**	**Nurse**	**Midwife**	**Total**
West Java	Ciamis	0.07	0.57	0.32	0.96

	Cirebon	0.14	0.37	0.34	0.85

	Garut	0.06	0.43	0.21	0.70

	Subang	0.12	0.54	0.32	0.97

	Sukabumi	0.09	0.26	0.18	0.54

Central Java	Brebes	0.10	0.35	0.32	0.77

	Cilacap	0.11	0.51	0.34	0.96

	Jepara	0.12	0.51	0.36	0.99

	Pemalang	0.10	0.39	0.23	0.72

	Rembang	0.16	0.56	0.72	1.43

East Java	Jombang	0.25	0.48	0.34	1.07

	Ngawi	0.15	0.52	0.24	0.91

	Pamekasan	0.11	0.38	0.32	0.82

	Sampang	0.07	0.36	0.21	0.64

	Trenggalek	0.11	0.52	0.32	0.95

These provider density levels are low by international standards and vary widely between districts. For example, the World Health Organization [3] defines 2.5 health care providers (doctors, nurses and midwives) per 1000 population as the level below which there is a critical shortage of providers. None of the 15 districts comes close to reaching the WHO cut-off – in fact, 11 of the 15 districts have densities below 1.0.

While these levels are undoubtedly low, the definition of density does not take into account the high level of dual practice that exists in many countries, including Indonesia. In fact, most health care providers practice twice, once at their position in the public sector and later in the day at their private practice. Taking this into account would undoubtedly raise the "provider" density but still not to the cut-off level suggested by WHO. At the same time, this effect is likely to overwhelmed by the high rates of absenteeism from public health centres, the site of the largest concentrations of health staff at the subdistrict level: an international survey showed Indonesia to have the highest rates of absenteeism for health staff (40%) across the countries surveyed [4].

### Service status

The employment status of health care providers in each district is summarized for doctors in Table 7, nurses in Table 8 and midwives in Table 9. (The actual frequencies in each of the employment status categories for each professional group are shown by district in Tables 10, 11 and 12 for West Java Province, Central Java Province and East Java Province, respectively.) The important points to arise from these tables are that in 2006:

**Table 7:** Distribution (proportion) of doctors by employment status and district in 15 districts, 2006

**Province**	**District**	**Permanent civil servant**	**Contract**	**Private practice**
West Java	Ciamis	0.52	0.08	0.40

	Cirebon	0.21	0.15	0.64

	Garut	0.41	0.22	0.37

	Subang	0.31	0.14	0.55

	Sukabumi	0.25	0.27	0.48

Central Java	Brebes	0.74	0.02	0.24

	Cilacap	0.48	0.15	0.37

	Jepara	0.65	0.33	0.02

	Pemalang	0.47	0.24	0.29

	Rembang	0.73	0.15	0.12

East Java	Jombang	0.41	0.09	0.51

	Ngawi	0.48	0.24	0.27

	Pamekasan	0.64	0.16	0.20

	Sampang	0.53	0.38	0.09

	Trenggalek	0.81	0.19	0.00

15 districts		0.46	0.17	0.37

**Table 8:** Distribution (proportion) of nurses by employment status and district in 15 districts, 2006

**Province**	**District**	**Permanent civil servant**	**Contract**	**Private practice**
West Java	Ciamis	0.51	0.44	0.04

	Cirebon	0.40	0.51	0.09

	Garut	0.58	0.42	0.00

	Subang	0.38	0.59	0.03

	Sukabumi	0.37	0.60	0.03

Central Java	Brebes	0.40	0.37	0.23

	Cilacap	0.52	0.30	0.18

	Jepara	0.71	0.26	0.03

	Pemalang	0.46	0.37	0.18

	Rembang	0.84	0.16	0.00

East Java	Jombang	0.32	0.38	0.30

	Ngawi	0.71	0.29	0.00

	Pamekasan	0.65	0.35	0.00

	Sampang	0.44	0.54	0.01

	Trenggalek	0.64	0.36	0.00

15 districts		0.51	0.41	0.08

**Table 9:** Distribution (proportion) of midwives by employment status and district in 15 districts, 2006

**Province**	**District**	**Permanent civil servant**	**Contract**	**Private practice**
West Java	Ciamis	0.68	0.25	0.07

	Cirebon	0.49	0.19	0.31

	Garut	0.59	0.37	0.04

	Subang	0.53	0.38	0.10

	Sukabumi	0.61	0.30	0.09

Central Java	Brebes	0.38	0.56	0.06

	Cilacap	0.66	0.34	0.00

	Jepara	0.63	0.37	0.00

	Pemalang	0.55	0.45	0.00

	Rembang	0.51	0.47	0.02

East Java	Jombang	0.50	0.31	0.19

	Ngawi	0.83	0.06	0.11

	Pamekasan	0.43	0.23	0.34

	Sampang	0.64	0.33	0.03

	Trenggalek	0.65	0.35	0.00

15 districts		0.56	0.34	0.10

**Table 10:** Distribution (frequency) of doctors, nurses and midwives by employment status and district in five districts of West Java, 2006

**District**	**Provider**	**PNS**	**PTT**	**Local contract**	**Volunteer/daily contract**	**Private sector**	**Total**
Ciamis	Doctor	50	3	0	5	38	96

	Nurse	430	0	161	210	34	835

	Midwife	320	107	5	7	33	472

	Total	800	110	166	222	105	1403

Cirebon	Doctor	62	39	6	0	188	295

	Nurse	322	0	335	72	71	800

	Midwife	357	106	3	30	227	723

	Total	741	145	344	102	486	1818

Garut	Doctor	59	22	11	0	53	145

	Nurse	573	0	295	116	0	984

	Midwife	277	110	17	46	18	468

	Total	909	132	323	162	71	1597

Subang	Doctor	53	16	8	1	95	173

	Nurse	289	0	274	168	20	751

	Midwife	233	146	10	10	43	442

	Total	575	162	292	179	158	1366

Sukabumi	Doctor	52	43	11	1	99	206

	Nurse	218	0	275	75	20	588

	Midwife	246	55	2	66	37	406

	Total	516	98	288	142	156	1200

**Table 11:** Distribution (frequency) of doctors, nurses and midwives by employment status and district in five districts of Central Java, 2006

**District**	**Provider**	**PNS**	**PTT**	**Local contract**	**Volunteer/daily contract**	**Private sector**	**Total**
Brebes	Doctor	134	3	0	0	44	181

	Nurse	238	0	223	0	138	599

	Midwife	206	306	1	0	35	548

	Total	578	309	224	0	217	1328

Cilacap	Doctor	88	23	5	0	67	183

	Nurse	453	4	261	0	155	873

	Midwife	389	107	89	0	0	585

	Total	930	134	355	0	222	1641

Jepara	Doctor	84	28	15	0	3	130

	Nurse	393	0	144	0	15	552

	Midwife	241	142	0	0	0	383

	Total	718	170	159	0	18	1065

Pemalang	Doctor	61	16	15	0	38	130

	Nurse	237	0	191	0	91	519

	Midwife	171	127	15	0	0	313

	Total	469	143	221	0	129	962

Rembang	Doctor	67	12	2	0	11	92

	Nurse	275	0	54	0	0	329

	Midwife	217	191	8	0	9	425

	Total	559	203	64	0	20	846

**Table 12:** Distribution (frequency) of doctors, nurses and midwives by employment status and district in five districts of East Java, 2006

**District**	**Provider**	**PNS**	**PTT**	**Local contract**	**Volunteer/daily contract**	**Private sector**	**Total**
Jombang	Doctor	122	17	9	0	153	301

	Nurse	185	0	154	63	175	577

	Midwife	203	106	22	0	77	408

	Total	510	123	185	63	405	1286

Ngawi	Doctor	64	0	14	18	36	132

	Nurse	316	0	130	0	0	446

	Midwife	169	0	8	4	22	203

	Total	549	0	152	22	58	781

Pamekasan	Doctor	56	12	2	0	17	87

	Nurse	193	0	106	0	0	299

	Midwife	109	40	17	0	87	253

	Total	358	52	125	0	104	639

Sampang	Doctor	28	7	13	0	5	53

	Nurse	129	0	101	57	4	291

	Midwife	110	48	7	1	5	171

	Total	267	55	121	58	14	515

Trenggalek	Doctor	59	11	3	0	0	73

	Nurse	230	0	128	0	0	358

	Midwife	140	71	5	0	0	216

	Total	429	82	136	0	0	647

• For all three professional groups (doctors, nurses and midwives) approximately half are permanent civil servants, or PNS (doctors 46%, nurses 51%, midwives 56%).

• Central government contracts (PTT) are of most importance for midwives (nine districts had more than one third of their midwives employed on this basis) and of declining importance for doctors. Nurses were not included in this scheme.

• Local contracts are most important for nurses (41% across the 15 districts).

• The private sector as the primary source of employment is most important for doctors (37% across the 15 districts): in four districts the proportion of doctors in the private sector was greater than the proportion of PNS. For midwives, the proportion is substantial: six districts had more than 10% of their midwives in private practice; in two of these districts approximately one third were in private practice. For nurses the proportion is low (8%), most in the private sector working in private hospitals.

### Primary place of work for those in the public sector

The database constructed for health staff in each province did not allow reliable differentiation on this variable in East Java. Consequently only West and Central Java are included here, a total of 10 districts. Health care providers at the district level whose primary place of work is in the public sector work in a limited number of institutions: doctors and nurses work in either the district hospital or a health centre; midwives work in the district hospital, a health centre or as a village midwife. The distribution across these public sector facilities is shown for doctors, nurses and midwives in Tables 13, 14 and 15, respectively. The main points to emerge from these tables are that in 2006:

**Table 13:** Distribution (proportion) of doctors working for the government by their primary place of work and district in 10 districts, 2006

**Province**	**District**	**Hospital**	**Health centre**
West Java	Ciamis	0.37	0.63

	Cirebon	0.21	0.79

	Garut	0.29	0.71

	Subang	0.38	0.62

	Sukabumi	0.34	0.66

Central Java	Brebes	0.33	0.67

	Cilacap	0.50	0.50

	Jepara	0.32	0.68

	Pemalang	0.30	0.70

	Rembang	0.35	0.65

10 districts		0.33	0.67

**Table 14:** Distribution (proportion) of nurses working for the government by their primary place of work and district in 10 districts, 2006

**Province**	**District**	**Hospital**	**Health centre**
West Java	Ciamis	0.20	0.80

	Cirebon	0.20	0.80

	Garut	0.40	0.60

	Subang	0.30	0.70

	Sukabumi	0.36	0.64

Central Java	Brebes	0.27	0.73

	Cilacap	0.32	0.68

	Jepara	0.20	0.80

	Pemalang	0.51	0.49

	Rembang	0.63	0.37

10 districts		0.32	0.68

**Table 15:** Distribution (proportion) of midwives working for the government by their primary place of work and district, 2006

**Province**	**District**	**Hospital**	**Health centre**	**Village**
West Java	Ciamis	0.03	0.44	0.52

	Cirebon	0.05	0.20	0.75

	Garut	0.04	0.28	0.69

	Subang	0.07	0.30	0.64

	Sukabumi	0.05	0.26	0.69

Central Java	Brebes	0.03	0.34	0.62

	Cilacap	0.06	0.51	0.43

	Jepara	0.03	0.57	0.40

	Pemalang	0.05	0.41	0.54

	Rembang	0.06	0.94	0.00

10 districts		0.05	0.41	0.54

• Overall, two thirds of doctors and nurses in the public sector are in the health centre and one third are in the district hospital.

• Overall, 54% of midwives were located at the village level, 41% were in the health centre and 5% in the district hospital. The proportion at the health centre is higher than expected and does not conform to the original intention of the village midwife programme. It is possible that these are recording errors, but checking of the records with district staff did not change the picture. On this basis, four districts have less than 55% of their midwives recorded as located at the village level.

## Discussion

The data presented here represent the stock and distribution of health personnel in 15 districts in 2006. In fact, since these data were collected, central government has been following up on an earlier promise to convert those on contract (including both PTT and local contracts) to permanent civil service status by the end of 2009; the major beneficiaries will be nurses on local contract and midwives on PTT. In some districts this could mean as many as 500 new permanent civil servants in the health sector. Consequently, the proportion of PNS will rise substantially and that for contracts will be much lower, essentially zero. Overall, there will be little change in the total number of providers, as those who convert to PNS are usually already on local contract or PTT.

Thus, PNS is still the most important employment category for all types of health care providers; contract employment (PTT and local contracts) is rapidly decreasing (although some form of local contract may increase again in the future as districts strive to get some flexibility back into their payrolls); most doctors and nurses are in the health centre; and the proportion of midwives in the village is less than expected. Private practice as the primary source of employment is now quite important, especially for doctors, and increasingly so for midwives. There is considerable variation between districts. Clearly, the distribution (both in aggregate and in any given district) between employment categories and facilities in 2006 is the outcome of various policies and actions taken in the last 30 years, policies and actions which have their origins in decisions taken 50 years ago as the post-Independence health system was planned and implemented.

There are four main points to make about these results. First, to explain the development of the human resource situation to this point; second, the emerging importance of the private sector, those not employed by the government; third, to assess the affect of decentralization; and fourth, to canvass where Indonesia goes from here.

First, the antecedents: In the late 1960s and early 1970s the government moved to set up a health system based on the health centre at the subdistrict level and a hospital at the district level [5]. The main goal was to improve access to health services under the umbrella of primary health care, an approach that was well under way in Indonesia by the time of the WHO- and UNICEF-sponsored Alma Ata conference [6].

It was agreed that to achieve this improved access, health facilities needed to be distributed among the people and the facilities needed to be adequately staffed. These two types of health facilities, district hospitals and health centres, were to be staffed by doctors, nurses and midwives. Subsequent decisions lead to health subcentres located in some villages, staffed by midwives and/or nurses and, even later, to the creation of a village facility staffed by midwives.

Once the basic structure of the health system was decided upon and under development, with health centres and hospitals being built, staffing the facilities became the critical activity. To do that, starting in the mid-1970s, the government introduced a period of obligatory service (as permanent civil servants or PNS, see Additional file. 1) for all new medical and nursing graduates. A period of obligatory service in places decided upon by the government allowed facilities to be established and staffed in many areas previously without health facilities, including some areas that were quite remote. The result was a rapid expansion of public health facilities and employment on the public payroll of the staff required to run them. Between the mid-1970s and the early 1990s essentially all doctors, nurses and midwives were employed in the public sector;. Because they were on the public payroll during this period (and the independent private sector was very small) the government potentially had basic information (age, sex, qualification and location) about nearly all human resources for health.

By the early 1990s the government realized that for fiscal reasons it could not continue to hire all new medicine, nursing and midwifery graduates and introduced a contract scheme (PTT – see Additional file. 2) for doctors and midwives (not nurses) that allowed them to meet their period of obligatory service (three years on Java, but shorter periods in more isolated areas) after which they could continue with specialist training and/or private practice. At the end of the 1990s the PTT system for doctors was under serious strain and finally ended in 2007, except for a small number of doctors serving for short periods (six months) in remote areas. The PTT system for midwives continues, with the intent of placing them in villages.

Second, the emerging private sector: Estimates for all categories of health care provider from this and earlier studies indicate a growing private sector [7,8], working either for private facilities (private hospitals, treatment clinics) or in their own private practice without an appointment with the government. Numbers from the current study are likely to be underestimates, as the membership lists and associated information kept by the professional societies are usually not up to date.

The flow of new graduates for each health care provider category has increased markedly in recent years as private training institutions have proliferated under a generally lax licensing approach. Now that PTT for doctors has effectively ended, and after the current PNS hiring phase, it is likely that, for fiscal reasons alone, few additional doctors, nurses and midwives will find employment with the government. Consequently, many, if not most, new graduates will move straight into private practice without any government position. So the proportion of private providers will certainly grow over the next decade and beyond if the current flow of new graduates continues.

The government has very limited and patchy information about providers who work only in private practice and are not on the government payroll: essentially they are not included in the Health Human Resources Information System, even in districts where the system is fully implemented. Governments ignore these trends (an increase in the proportion of providers in private practice and the decrease in what is known about them) at their peril, as the private sector is an important source of health care and is likely to become more so in the future. Further, these private providers are likely to be politically active and have great potential to skew the further development of the health system in ways that will put delivery of health public goods, and the poor, at even further disadvantage than is currently the case.

Third, the effect of decentralization: What is clear is that, despite assertions to the contrary, decentralization initially brought no increase in "decision space" about human resources for health in the districts. Although public sector health staff were "transferred" to the districts under decentralization, the reality is that the centre retained control over salaries, conditions and hiring and firing: the decision space for districts about these staff was essentially zero before decentralization and did not change afterwards.

In an effort to create some flexibility in their hiring, districts had started to place greater reliance on local contract hiring even before decentralization and, in some districts, this had become more important in the first years of decentralization: for a brief moment the decision space widened. Now even that avenue of flexibility has been closed by the centre with their conversion to PNS of those currently on contracts (either central or local).

Beyond that, further contract hiring by the districts has been forbidden, so this window has been closed: almost all staff are now fixed costs and decision space for districts about staff reduced to virtually zero. Further, the salaries for all PNS is the first charge against the so-called unconditional grant from the central government, further reducing their overall decision space on the sector budget. Decentralization, then, has actually decreased the decision space of the district with respect to human resources, which account for as much as 40% of district expenditure on health [Heywood P, Harahap NP: Public spending on health at the district level in Indonesia after decentralization – sources, flows and contradictions, unpublished.] the single largest item in their budget. A related consequence is that by assuming control of all human resources for health, the central government once again has reduced pressure on district governments to allocate public resources efficiently [7].

Experience from various countries indicates that decentralization may have additional implications for human resources, including in the health sector [9]. These vary from the deterioration of human resource databases, the need for new skills at all levels, confusion and conflicting goals for human resource management at different levels of the system and effects on training and staff mobility, to the need for local managers to have flexibility in their labour costs and the space to change the skill mix of their staff. All these effects are evident in Indonesia.

No doubt this move by the central ministry to regain control of public sector human resources for health potentially means it will have better information about those providers who are on the public payroll. However, unless there is a radical change in the Health Human Resources Information System there will be no improvement in information about the increasingly important private sector: effectively the government information about human resources for health in the sector as a whole will decrease.

Fourth, what of the future? The Indonesian National Health System, as it evolved, had an implicit aim of distributing health care providers throughout the country. The key to achieving this distribution was the establishment of a network of publicly funded health care facilities in which the central institution was the public health centre, a centre that was also seen as consistent with implementation of the Health For All goals of the Alma Ata declaration [6].

Facilities were established in a fixed ratio to the population. Facilities required a fixed complement of staff. All doctors, nurses and midwives had to work for the government. There was no independent private sector. Thus, estimating human resource requirements was a simple arithmetic exercise and was not related to local variations in workload or overall efficiency of resource use. Training institutions had to provide these staff.

This simple, linear logic was the essence of the human resources policy of the government right through the Suharto era. A new category of staff (the midwife) could be added, subtracted and then added again; new forms of employment were devised (the PTT scheme) and modified as new problems arose. But essentially, implementation of the policy involved a series of calculations to estimate the number of staff required to run the facilities that had been, or were to be, built.

For a period, at the height of the Suharto era, this approach worked. Facilities and staff were basically distributed as planned, services were delivered and health status improved. Whether this improvement was due to the health facilities and staff or to the economic development, improving basic education and road infrastructure, or the poverty reduction that occurred at the same time, is still an open question [7,10,11].

But after this brief heyday in the second half of the 1980s the system began to slowly, but surely, unravel. And the main reasons for this were problems with human resources. Service providers create and deliver the service and are usually seen by the consumer as synonymous with service quality [12]. The quality of training had always been low and there was little improvement as the pressure to train and graduate more providers to staff the growing number of facilities increased.

A crucial underlying problem was the inability of the government to maintain the relatively good distribution of staff it achieved in the latter part of the 1980s. Contributing to this initial success was that many of the health staff trained at the end of the 1970s and the first part of the 1980s were highly motivated and dedicated people who genuinely wanted to bring health care to their less-fortunate compatriots.

There is also no doubt that many had no real interest in their fellow nationals beyond making a comfortable living. The best way to do that was to establish a successful private practice; in the early years this was in addition to compulsory public sector employment, but more recently is completely independent of the government. The best location usually was, and still is, in urban areas. In Indonesia, as in most other countries, the resting state for the distribution of health care providers is to be located in urban areas. While this applies more to doctors than to the other professions, nurses and midwives also have many of the same motivations and also tend to gravitate to urban areas [13]. Further, as the student nurses and midwives are increasingly drawn from families of the urban middle class, the preferences for urban living increase.

The issue for any government now, as it was 50 years ago when the current system was devised, is how to improve the quality of health services and ensure access to them. Human resources are crucial to this effort. Under the assumption that the task still is to distribute facilities and providers to the people, to a large extent the government has lost the most potent tool it had in the 1970s and 1980s to improve distribution: coercion of health care providers to serve the government where the government wanted them to.

Further, Indonesia is now a more urbanized country with a much higher level of income as well as much lower, though persistent, levels of poverty; education levels have increased and road infrastructure has also improved. In effect the population overall has more money, is better educated and more mobile. Even if the poverty, education and infrastructure situations are still far from ideal, they are much better than they were 50, or even 30, years ago. The epidemiological transition, together with the demographic changes that have taken place, mean that the problems faced by the health system have changed dramatically in favor of noncommunicable diseases.

The challenge now is not to devise new ways to continue implementation of the old health system. The challenge now is to develop a vision for a new health system that takes these contextual changes into account as it addresses the changed health needs. In envisioning the new health system, the government and community will need to take account of the difficulty the government now has in maintaining the distribution of health care providers that characterized the Suharto era, as well as recognize that managing the whole system requires more complex management skills and includes taking into account that private providers are an increasingly important alternate source of care [14,15]. The situation is further complicated by the fact that the government has inadequate information about the stock of health care providers at the district level, especially the rapidly increasing private sector, and limited capacity for human resource planning at all levels.

But the first step is establishing a new vision, something that the current government has been unable to do. When the new vision is there, then the questions of how to ensure that there are health care providers and facilities of quality in the best places to ensure reasonable access and an improved health situation for the Indonesia of today and, more importantly, tomorrow, can be addressed.

## Conclusion

The Indonesian health system has its origins in decisions taken 50 years ago as the post-Independence health system was planned and implementation commenced. The main goal was to improve access to health services under the umbrella of primary health care. This was done by establishing and staffing a network of primary care facilities based around a health centre in each subdistrict and a hospital in each district. Starting in the mid-1970s, these facilities were staffed through a period of obligatory government service for all new graduates in medicine, nursing and midwifery. By the mid-1990s this was no longer possible for fiscal reasons and a contract system of up to five years, depending on the location of service, was implemented. This system ended in 2007 with provider density still low by international standards. In effect, the government has now lost its most potent tool to improve distribution of providers – obligation to serve the government where the government wanted – and has been unable to replace it.

Under decentralization, districts were to have greater control over public sector human resources at the district level and would, thereby, have an incentive to make more efficient use of them. In fact, the central government retained control over most public sector human resources. Immediately before and after decentralization, district governments increased their use of local contract staff as a way of gaining flexibility in their wage bill and skill mix. However, central government has recently moved to regain control over essentially all public sector staff by converting PTT and contract staff to permanent civil servants. In effect, Indonesia has returned to the centralized control over public sector human resources of 20 years ago.

However, during the last decade the private sector expanded rapidly because the government uptake of new graduates is much lower than previously; the majority now enter the private sector directly. The government has little information about the private sector, which is already substantial, particularly for doctors, and will increase in both size and importance in the coming decade.

At the same time, Indonesia has changed dramatically since the existing health system was designed. It is now more urban, has higher incomes and less poverty, better education and is more mobile. The health situation has also changed: even though communicable diseases remain important, noncommunicable diseases are now dominant. The challenge now is to envision a new health system that takes into account the changing context as it addresses the new health needs of the nation. Envisioning the new system is a crucial first step for development of a human resources policy which, in turn, will require more information about the stock of health care providers, private as well as public, and increased capacity for human resource planning.

## Competing interests

The authors declare that they have no competing interests.

## Authors' contributions

PH conceived the study, analysed results and drafted the manuscript. NPH provided input on study design, supervised data collection in West Java Province, assisted with interpretation of results and reviewed the manuscript.

## Supplementary Material

Additional file 1 **Supplementary file 1**. Permanent civil servants (*Pegawai Negeri Sibil *– PNS) [7,8,13,15] . Click here for file

Additional file 2 **Supplementary file 2.** Central contracts (*Pegawai Tidak Tetap *– PTT) [13,15]. Click here for file
