# Supplementary material for: Human resources for health at the district level in Indonesia: the smoke and mirrors of decentralization
Source: Hum Resour Health. 2009 Feb 3;7:6. doi: 10.1186/1478-4491-7-6 (PMC2662783; doi:10.1186/1478-4491-7-6)
Supplement: Additional file 1 — Supplementary file 1. Permanent civil servants (Pegawai Negeri Sibil – PNS) [7,8,13,15] . [file 1478-4491-7-6-S1.pdf]

Persons appointed as PNS are permanent civil servants. Under a 1974 Presidential Decree, all new medical graduates were required to enter government service as permanent civil servants (PNS). There was a period of obligatory service at a health facility, usually a health centre, nominated by the government. Fulfilment of this obligation was a condition of obtaining a licence to practise. The length of mandatory service, normally five years, was reduced to three years in areas outside Java and two years in remote areas such as Irian Jaya. In addition to the usual civil service benefits, health centre doctors received free housing. After the period of compulsory service (*masa bakti*), doctors could then apply to enter specialist training or be assigned to another location or stay where they were. While serving at the health centre, the doctor was permitted to engage in private practice outside government working hours. Between 1974 and 1992, a total of 8327 doctors were recruited in this way and assigned to health centres; a smaller number were assigned to hospitals. Nurses, and later midwives, were also recruited as permanent civil servants.

By the late 1980s it was becoming clear to the government that for budgetary reasons it would not be able to continue to take all doctors and nurses as permanent civil servants with the attendant commitments to health care, pensions and other benefits. In 1992 the government introduced a zero-growth policy for the whole civil service. Recruitment of new PNS doctors was limited to those required to replace doctors lost through retirement, death and migration. Within the health sector the response was to develop a category of contract workers (PTT – *Pegawai Tidak Tetap*) for doctors and, subsequently, midwives, whose period of obligatory service was handled through a non-renewable three-year appointment to serve in remote areas – see Fig. 2. Nurses were not included in this new category. New recruits to the permanent civil service were limited in number.

This policy of limited new recruits to the permanent civil service continued through 2005, when the central government decreed that all central and local contract appointees up to that time who met certain age and length-of-service criteria would be converted to permanent civil servants. The criteria were subsequently relaxed so that all persons 19 to 46 years of age who had served at least one year on a central or local contract would be eligible for appointment as a permanent civil servant before the end of 2009.
