# Supplementary material for: Human resources for health at the district level in Indonesia: the smoke and mirrors of decentralization
Source: Hum Resour Health. 2009 Feb 3;7:6. doi: 10.1186/1478-4491-7-6 (PMC2662783; doi:10.1186/1478-4491-7-6)
Supplement: Additional file 2 — Supplementary file 2. Central contracts (Pegawai Tidak Tetap – PTT) [13,15]. [file 1478-4491-7-6-S2.pdf]

In 1992, as part of the overall government policy of limiting civil service growth, newly graduated doctors were no longer hired as permanent civil servants (PNS). However, there was still a requirement for obligatory service and this was now fulfilled through employment on a three-year contract as non-permanent employees (*pegawai tidak tetap* – PTT). Employment as a PTT doctor was a condition of obtaining a licence to practise. The salary and allowances received by PTT doctors were far more attractive than those of permanent civil servants; however, PTT doctors did not receive health care, housing, pension and other benefits received by PNS doctors. Doctors were assigned mostly to health centres.

The rhetoric surrounding the scheme emphasized the intention to improve the distribution of doctors to rural and remote areas. The PTT scheme faced administrative issues from the start, especially the inability of the government to place all new graduates at the start of each year. As a result, many young doctors had to wait until their placements became available and frequently worked as unlicensed doctors in urban private practices. The compulsory nature of the service was questioned; salaries were considered low, especially for graduates from private medical schools where the cost of training was much higher; some considered restrictions on choice of location infringed human rights; many of the regulations could be avoided at a price; and, despite the scheme, concentration of doctors on Java and in urban areas continued.

With each new set of issues and criticisms the government modified the scheme, sometimes by increasing pay, at other times by softening the conditions of service. Before the scheme for doctors was eventually abandoned in 2007, the conditions had been modified so much that new doctors faced few limitations that could not be negotiated away. Today what remains of the scheme for doctors is a voluntary six-month contract to serve in remote areas with substantially higher salary and location allowances than PNS doctors. For all non-remote areas, the central PTT scheme for doctors has been terminated.

However, the PTT scheme still operates throughout Indonesia for midwives, for whom the scheme also started operation in 1994. It was used to drive the village midwife scheme. Midwives were hired on a non-permanent basis for three years, with a maximum extension of six years. It was assumed that midwives who had completed their contract would be hired as PNS or take up private practice or continue their education. As with the doctors, some aspects of the scheme were found to be untenable. The main problem was the limitation on extensions, which was subsequently lifted. At the same time, there are concerns in some districts that many midwives are no longer in the villages but have moved to urban areas, where more lucrative private practices can be established, while retaining a nominal village location. This concern comes at a time when the central government is engaged in a major expansion of the midwife scheme in two ways. First, as with other health staff, PTT midwives who were employed before 2005 have already been offered the chance to become permanent public servants. Second, the central government in 2008 asked district health offices to send names of midwives to be appointed as new PTT midwives. To date there has been no physical field-based census of midwives, PNS or PTT, to determine if they are in fact in the village.
